# Stage effects of negative emotion on spatial and verbal working memory

**DOI:** 10.1186/1471-2202-11-60

**Published:** 2010-05-07

**Authors:** Xuebing Li, Raymond CK Chan, Yue-jia Luo

**Affiliations:** 1 Key Laboratory of Mental Health, Institute of Psychology, Chinese Academy of Sciences, Beijing, China; 2 Neuropsychology and Applied Cognitive Neuroscience Laboratory, Institute of Psychology, Chinese Academy of Sciences, Beijing, China; 3 State Key Laboratory of Cognitive Neuroscience and Learning, Beijing Normal University, Beijing, China

## Abstract

**Background:**

The effects of negative emotion on different processing periods in spatial and verbal working memory (WM) and the possible brain mechanism of the interaction between negative emotion and WM were explored using a high-time resolution event-related potential (ERP) technique and time-locked delayed matching-to-sample task (DMST).

**Results:**

Early P3b and late P3b were reduced in the negative emotion condition for both the spatial and verbal tasks at encoding. At retention, the sustained negative slow wave (NSW) showed a significant interaction between emotional state and task type. Spatial trials in the negative emotion condition elicited a more negative deflection than they did in the neutral emotion condition. However, no such effect was observed for the verbal tasks. At retrieval, early P3b and late P3b were markedly more attenuated in the negative emotion condition than in the neutral emotion condition for both the spatial and verbal tasks.

**Conclusions:**

The results indicate that the differential effects of negative emotion on spatial and verbal WM mainly take place during information maintenance processing, which implies that there is a systematic association between specific affects (e.g., negative emotion) and certain cognitive processes (e.g., spatial retention).

## Background

The modulation of cognition by emotion is of considerable interest both theoretically and clinically. Processing efficiency theory suggests that the effects of negative emotion (e.g., anxiety) on cognitive performance may be mediated by their effects on working memory (WM) [1]. WM refers to a system used for the temporary storage and manipulation of information that is deemed necessary for a range of more complex cognitive activities. Baddeley [2] hypothesized that WM is composed of three major components that work independently of one another: (i) a verbal WM system for tackling verbal information in the phonological loop; (ii) a visuospatial WM system for processing non-verbal information in the visuospatial sketchpad; and (iii) a central executive that determines the information that is made available for conscious processing by exerting control over voluntary action. Eysenck and Calvo proposed that the major effects of anxiety (e.g., worry) are exerted on the modality-free central executive, which initiates a chain reaction on the verbal and spatial components of WM and ultimately disrupts both systems [1,3]. However, in contrast to the general effect of anxiety on the central executive, some clinical studies have shown that people with anxiety or depression disorders have specific deficits in verbal or visuospatial memory, which implies that the two WM systems are disrupted unequally by negative effects. For example, obsessive-compulsive disorder (OCD) appears to be associated with deficits in visual or visuospatial memory, but not with verbal WM [4]. Depression disorders are linked to a specific deficit in the performance of spatial tasks, but have no significantly different effect on verbal performance compared with controls [5-7]. In addition, a high trait anxiety results in worse performance in verbal WM than in spatial WM, with the opposite being the case for low trait anxiety [8].

Although the aforementioned research relies mainly on studies of mental disorders, more recent evidence obtained from healthy people is also consistent with the possibility that the effects of negative emotion on verbal WM may not be the same as those on visuospatial WM [9-12]. For example, Lavric and colleagues [9] employed threat-of-shock to induce anticipated anxiety in college students, and found that threat-evoked anxiety selectively impaired the performance of spatial n-back tasks but not verbal n-back tasks. In an electrophysiological study, Li et al. [10] used aversive and neutral pictures from the International Affective Picture System (IAPS) to induce intended emotion, and found reduced amplitudes in the anterior late positive component and the posterior P300 during negative emotion, with greater effects during spatial WM tasks than verbal WM tasks. These findings suggest that spatial and verbal WM may be differentially affected by induced negative emotion, and that spatial WM may be more vulnerable to negative emotion in healthy people. However, the neural mechanism that underlies this selective modulation is not yet clear.

Lavric and colleagues [9] proposed that threat-evoked anxiety mood and spatial WM rely on a common visuospatial attention mechanism. Because negative affect commonly draws visuospatial attention (e.g., attention to threat), which is also essential to spatial WM [13-15], there is an attentional resource competition between negative affect and spatial WM. In contrast, this competition is less pronounced when it comes to verbal WM due to its primary dependence on phonological processes [16,17]. Thus, visuospatial attention may overlap both negative emotion and spatial WM, which may result in induced selective impairment of spatial WM. This is an effective and reasonable hypothesis, but there is no further evidence to support it. Hence, based on the hypothesis that visuospatial attention is a factor that underlies this selective effect, it is argued that the effect should occur mainly during the phase of information retention, but not during the phases of encoding and retrieval in WM. As with long-term memory systems, WM involves three processes: encoding, retention, and retrieval. Encoding is the process of getting information into the memory, and depends on elaboration, which refers to how extensively information is processed at any given depth in the memory. Retrieval is the process of getting information out of the memory, and is influenced by the presence of cues and the nature of the task (e.g., recognition or recall). Much of the interest in retrieval has focused on long-term memory. Retention is the storage of information over time and the representation of information in the memory [18]. Previous studies have found that information representation at retention in WM is type specific, which means that spatial and verbal information representations are linked to different cognitive strategies and neural mechanisms [19,20]. As mentioned, the retention of spatial information relies on spatial selective attention and processes in the visuospatial sketchpad, whereas the retention of verbal information depends on articulatory rehearsal and manipulation in the phonological loop [21,22].

The purpose of this study was to further investigate whether negative emotion induced in healthy people is associated with selective impairment in spatial WM compared with verbal WM, and whether the nature of the impairment is consistent with a retention deficit. A delayed matching-to-sample task (DMST) was adopted that comprised three phases: target, delay, and probe. Distinct brain processing procedures can be separated by the three phases with this paradigm: information encoding in the target phase, information retention in the delay phase, and information retrieval in the probe phase [23]. An event-related potential (ERP) technique with sub-second resolution was used to make inferences about the role of different ERP components in processing procedures in WM.

Previous ERP studies have shown that the P3b subcomponent of P300 is strongly associated with memory processes. In earlier studies of long-term memory, researchers found that P3b and other late positivities that overlap with P3b varied as a function of subsequent recognition/recall performance. Usually, the P3b elicited by stimuli was larger for stimuli that were subsequently recalled than for stimuli that were not recalled. In most of these studies, the variations in ERP activity were observed in the 300-1200 ms latency range, and were thus probably associated with memory retrieval operation [24].

Later studies of ERP activity and short-term memory found that P3b was also elicited by memory sets during the period of encoding [22,25,26]. Ruchkin et al. [27] attempted to elucidate the ERP components associated with information storage and retention in short-term memory. They designed a memory and visual research task and analyzed the ERP components evoked by target stimuli. They found that neither latency nor average amplitude of P3b differed as a function of whether the task involved memory or a visual search. It was thus concluded that P3b is sensitive to information acquisition operations and does not appear to be specifically associated with memory retention. P3b was also interpreted as the "closure" of perceptual events processing during the encoding phase [28].

Morgan et al. [26] employed the DMST with faces and analyzed the ERP in both the encoding and retrieval phases. They found that P3b was evoked by both the target and the probe stimulus. At encoding, the P3b was divided into two subcomponents, early P3b (around 354 ms) and late P3b (around 512 ms). At retrieval, P3b was over the parieto-occipital electrodes, and divided into two peaks at 356 ms and 550 ms. A growing corpus of evidence shows that the P3b may consist of different subcomponents that reflect functionally and anatomically distinct neural processes [29]. This is especially the case when a more complex task is used. For example, in a study that used a delayed recognition task in which either one or three novel objects were sequentially presented for memory encoding, and then after a delay of several seconds a test stimulus requiring a match/mismatch response appeared, the P3b divided into two peaks with different neural generators [26]. The early P3b subcomponent (366 ms) was generated in the inferior temporal cortex, the left temporo-parietal junction, and the posterior parietal cortex (PPC), whereas the later subcomponent (585 ms) was generated mainly in the PPC and the ventrolateral prefrontal cortex [26,30]. Bledowski et al. [30] proposed that the early P3b subcomponent (356 ms) is related to stimulus evaluation processes, whereas the later subcomponent (550 ms) may reflect memory search processes.

Several studies have attempted to elucidate the ERP activity related to information storage and retention in WM. One long-duration slow wave was found that specifically indexed sustained mnemonic operations during the retention phase [31]. For example, Ruchkin et al. [32] asked subjects to memorize several items and probed their memory for the items after a 5000 ms blank interval. ERPs were time locked to the onset of the memory array and continued throughout the retention interval. During this interval, a negative slow wave (NSW) was observed over all of the brain scalp sites and was sustained throughout the retention period. The amplitude and scalp topography of the NSW has been found to vary as a function of the nature of the specific memory task, and several groups have used the NSW to measure spatio-temporal activation patterns during WM tasks. For example, Mecklinger and Pfeifer [19] found that the scalp distribution of the NSW is different for spatial and object DSM tasks. When spatial information was maintained in WM, the NSW activity rapidly rose at recording sites overlying the posterior parietal and occipital cortical areas. At these recording sites, the NSW increased in amplitude with increasing spatial memory load. In terms of object information, load-sensitive NSW activity was focused on the mid-frontal recording sites. These results provide evidence for the notion that retention processes for different types of WM may be functionally dissociated and involve differential patterns of neuronal activation.

The present study had two main goals. The first was to examine the selective effect of induced negative emotion on spatial WM. The second was to determine the time course of this selective effect in WM. A DMST was employed that allowed separate ERP investigation of the encoding, retention, and retrieval phases. Based on the results of previous work, the P3b elicited by the target stimuli and the probe stimuli were separately regarded as reflections of encoding and retrieval, and were expected to contain two subcomponents each. It was predicted that the amplitudes of the P3b subcomponents at encoding and retrieval would decrease in both spatial and verbal WM under a condition of negative emotion compared with a condition of neutral emotion. However, the NSW at retention was expected to indicate the selective influence of negative emotion on spatial WM, but not on verbal WM. Previous research has shown the average amplitude of the NSW to increase with increasing task difficulty [19]. In this study, it was hypothesized that the presence of negative emotion would increase the task difficulty relative to the neutral condition, and thus the amplitude of the NSW under the influence of negative emotion would be larger than that under the influence of neutral emotion.

## Results

### Subjective Ratings

The pictures induced the expected emotional states based on self-reported changes in emotion. Paired t tests between the baseline and post-session PANAS scores were conducted on the positive and negative PANAS sub-schedules in all of the subjects. After the aversive experimental session, the negative PANAS scores increased (t (14) = 3.98, p = 0.001) and the positive PANAS scores significantly decreased (t (14) = -3.25, p = 0.006). However, there were no overt changes in the sub-schedule scores after the neutral experimental session.

### Behavior

A repeated measures analysis of variance (ANOVA) of response times and accuracy was conducted with two factors: emotional state and task type. No significant main or interactive effects were identified for response times or accuracy. However, there was a tendency for a longer RT and better performance accuracy in the verbal tasks than in the spatial tasks (see Table 1).

**Table 1:** Means and standard deviations of response time (RT) and accuracy

Emotional state	Task type	RT (ms)	Accuracy (%)
Negative emotion	Spatial WM	759 ± 110	89.1 ± 6.8
	Verbal WM	802 ± 118	90 ± 5.4
Neutral emotion	Spatial WM	776 ± 110	87.7 ± 8.7
	Verbal WM	805 ± 119	90.9 ± 4.5

### Electrophysiology

#### Target stimulus-locked ERPs

##### Early P3b

**Figure 1:**
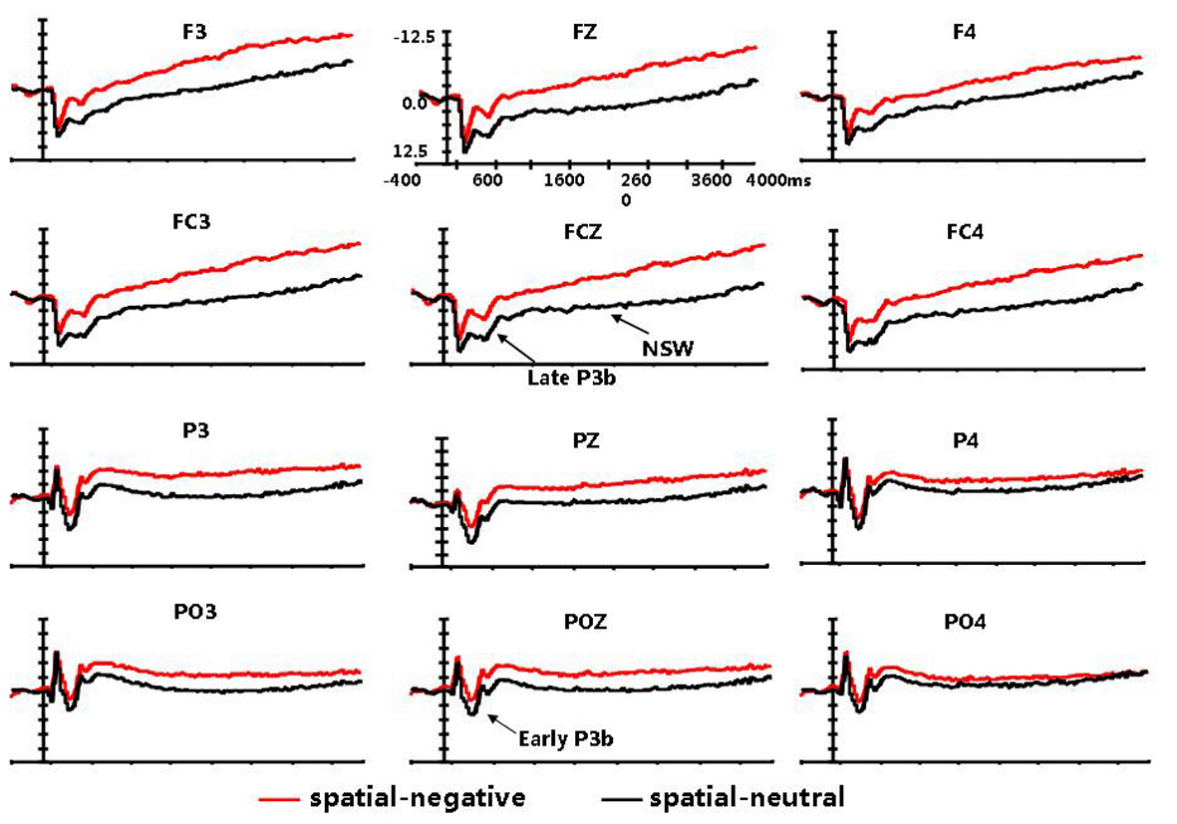
**Grand average ERPs for the target stimuli in the negative emotion and neutral emotion conditions at the 12 electrode sites. **Only the waveform of spatial WM is presented. Early P3b, Late P3b, and NSW are indicated on the waveform plots.

**Figure 2:**
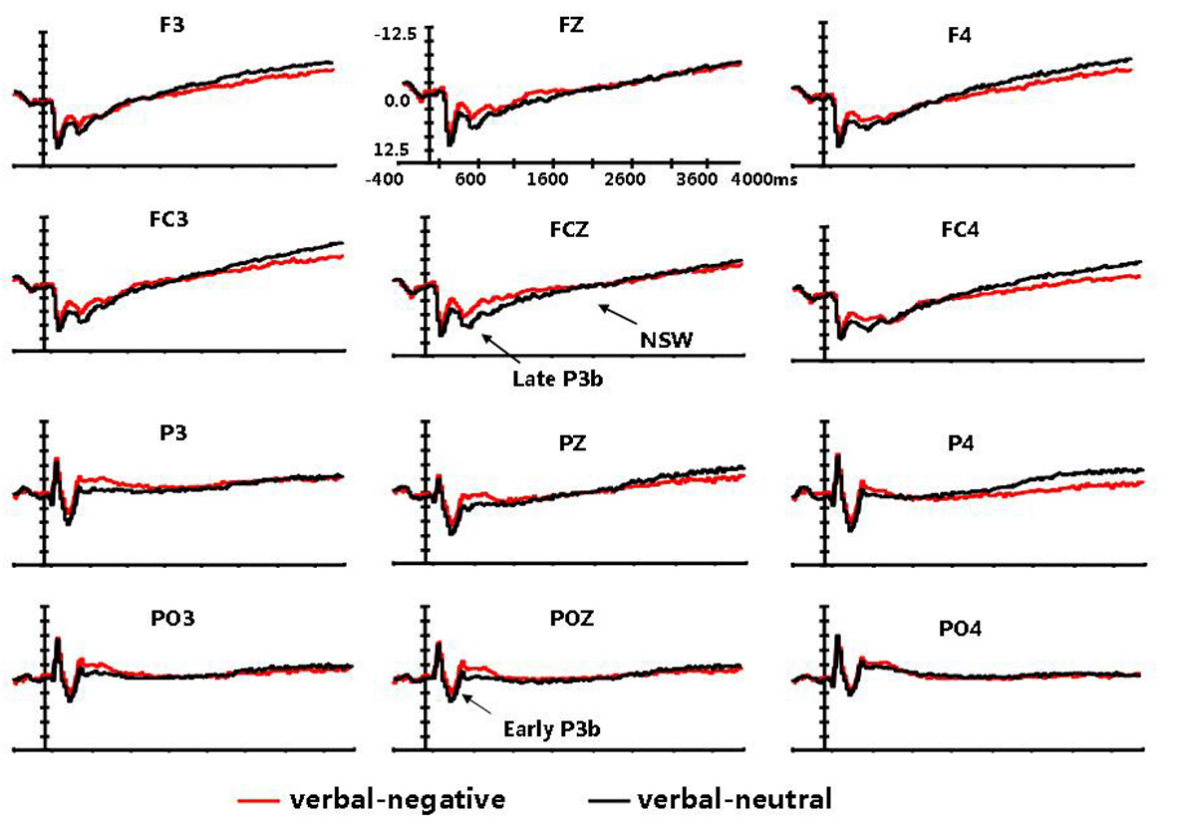
**Grand average ERPs for the target stimuli in the negative emotion and neutral emotion conditions at the 12 electrode sites**. Only the waveform of verbal WM is presented.

A four-way ANOVA of the mean amplitudes of early P3b measured at the posterior electrodes revealed significant main effects of emotional state (F(1,14) = 17.728, p = 0.001), laterality (F(2,28) = 4.418, p = 0.03), and anterior-posterior electrodes (F(2, 28) = 48.436, p < 0.001). There were also significant interaction effects of emotional state × laterality (F(2,28) = 4.346, p = 0.037), emotional state × anterior-posterior electrodes (F(2, 28) = 31.231, p < 0.001) and laterality × anterior-posterior electrodes (F(4, 56) = 7.074, p < 0.001). The comparisons of the effects of emotional state at each level of task type, laterality, and anterior-posterior electrodes showed the early P3b amplitudes in the negative emotion condition were smaller than those in the neutral emotion condition for both the spatial and verbal tasks (F(1,14) = 14.286, p = 0.002; F(1,14) = 8.2596, p = 0.012). This emotional state effect was larger over the midline and parietal electrodes than over the left-lateral and occipital electrodes (F(1,14) = 9.452, p = 0.008; F(1,14) = 75.233, p < 0.001). Overall, early P3b amplitude was largest at the PZ electrode (see Figures 1 and 2).

##### Late P3b

**Figure 3:**
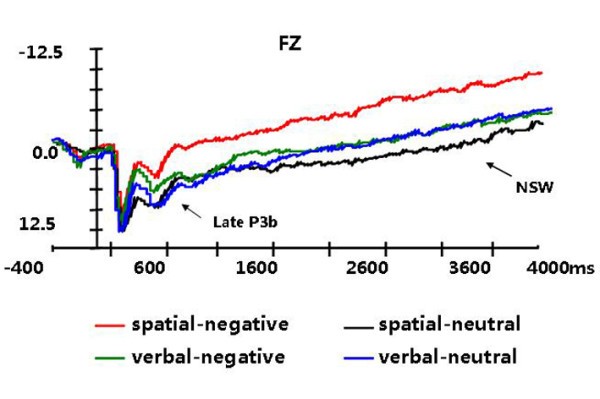
Grand average ERPs for the target stimuli in the negative emotion and neutral emotion conditions at Fz, and the waveforms of spatial and verbal WM.

An ANOVA of the mean amplitudes of late P3b measured at the anterior electrodes revealed a significant main effect of emotional state (F(1,14) = 38.151, p < 0.001), a main effect of anterior-posterior electrodes (F(3,42) = 13.977, p < 0.001), and an interaction effect of task type × emotional state (F(1,14) = 10.39, p = 0.006). The main effects showed that the amplitude of late P3b in the negative emotion condition was smaller than in the neutral emotion condition for both the spatial and verbal tasks, with the amplitudes increasing along the anterior-posterior dimension and maximal at the central electrodes (see Figures 1 and 2). The task type × emotional state interaction effect was examined by comparing the effects of emotional state at each level of task type and then comparing the effects of task type at each level of emotional state. The verbal tasks elicited larger late P3b amplitudes than the spatial tasks in the negative emotion condition (F (1, 14) = 9.467, p = 0.008), but not in the neutral emotion condition (F (1, 14) = 0.197, p = 0.664) (see Figure 3).

##### NSW

Sustained negative slow wave shifts showed condition-related differences over the whole scalp region. The mean amplitudes of the NSW measured at the 21 electrodes indicated an emotional state main effect (F(1,14) = 3.404, p = 0.086), laterality main effect (F(2,28) = 3.082, p = 0.062), emotional state × laterality interaction effect (F(2,28) = 7.169, p = 0.006), and emotional state ×task type interaction effect (F(1,14) = 17.33, p = 0.001). As with early and late P3b, further analysis of the interaction effects revealed the more important result that spatial tasks in the negative emotion condition elicited a more negative deflection than in the neutral emotion condition (F (1, 14) = 13.98, p = 0.002). However, this effect was not observed for the verbal tasks (F (1, 14) = 1.08, p = 0. 317) (see Figures 1 and 2). This emotional state effect was broadly distributed across the whole scalp region, but was relatively larger at the midline electrodes.

#### Probe stimulus-locked ERPs

##### Early P3b

**Figure 4:**
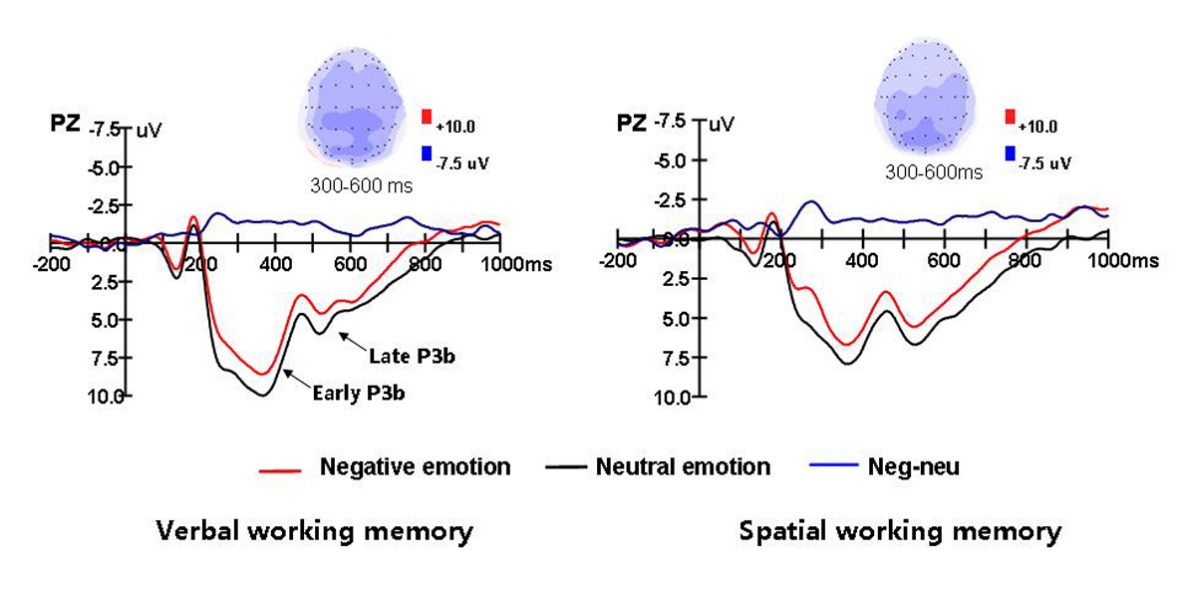
**Grand average ERPs for the probe stimuli in the negative emotion and neutral emotion conditions, the difference wave (negative minus neutral) at Pz, and topographic voltage maps of the difference waves at time windows of 300-600 ms.** The waveforms of spatial and verbal WM are presented separately.

There was a significant main effects of emotional state (F (1, 14) = 7.425, p = 0.016), a main effect of task type (F (1, 14) = 14.1, p = 0.002), a main effect of laterality (F (2, 28) = 3.036, p = 0.086), and a main effect of anterior-posterior electrodes (F (2, 28) = 31.918, p < 0.001) for early P3b. No significant interactive effect of emotional state and task type was found. Further analysis found that the probe stimuli in the negative emotion condition elicited a more negative shift than in the neutral emotion condition during an interval of between 230-460 ms, and that the verbal tasks elicited larger amplitudes for early P3b than the spatial tasks. The early P3b amplitudes were relatively larger at the midline and the parietal electrodes (see Figure 4).

##### Late P3b

There were significant main effects of emotional state (F(1, 14) = 3.778, p = 0.072), laterality (F(2, 28) = 7.947, p = 0.002), anterior-posterior electrodes, F(2, 28) = 13.221, p = 0.001), and the emotional state × anterior-posterior electrodes interaction (F(2, 28) = 5.717, p = 0.024). Overall, the late P3b amplitude was larger at the midline and parietal electrodes than at the lateral and occipital electrodes. The emotional state × anterior-posterior electrodes interaction effect was examined by comparing the effects of emotional state at each horizontal axis of electrodes (P/PO/O). Emotional state had a significant effect over the P and PO electrodes (F (1, 14) = 4.854, p = 0.045; F (1, 14) = 5.426, p = 0.035), but not over the O electrodes (F (1, 14) = 0.214, p = 0.651) (see Figure 4).

The topographic voltage maps of the difference waves revealed that the emotional state effects were mainly distributed over the posterior scalp in both spatial and verbal WM, which is the reason why only the posterior sites were analyzed in the probe phase (see Figure 4).

## Discussion

The aim of this study was to assess the effects of negative emotion on WM tasks. The effect of negative emotion was investigated for both spatial and verbal WM to determine whether they were general to WM processes, or specific to the type of WM task. We assessed the emotional effects during encoding, retention and retrieval periods of WM. Both early and late P3b components showed reduced amplitudes under the negative emotion condition compared with the neutral condition for both spatial and verbal WM tasks. However, the NSW component showed a greater negative shift only for the spatial WM task in the negative emotion condition. The electrophysiology results give some support to our hypothesis that the selective effect of negative emotion on spatial WM occurs during the period of information retention.

According to the self-reported data, the experimental procedure induced a relatively high level of negative emotion. The behavioral data did not show a significant emotional state effect, but indicated that the subjects responded faster and less accurately to the probe stimuli in the spatial tasks than in the verbal tasks, suggesting the use of different response strategies and neural mechanisms in these two types of WM, which is consistent with previous studies [19,20]. The absence of an emotional effect is possibly because the negative emotion evoked by the aversive pictures was not sufficiently strong to induce changes in behavioral performance. Fortunately, the ERP results give more information about the mental activity in response to the pictures.

First, the target stimuli-locked early and late P3b potentials showed reduced amplitudes under the negative emotion condition for both the spatial and verbal WM tasks. The P3b peaks, which reached maximum amplitude over the central-parietal region, occurred shortly after the presentation of the target stimuli and several hundred milliseconds before a response was executed. The time course suggests that these signals may reflect encoding and stimulus evaluation processes, rather than activity specifically related to the maintenance of temporary representations in WM. The results suggest that the encoding processes of both spatial and verbal WM are affected by negative emotion. Previous studies have shown that P3b amplitudes are sensitive to the allocation of processing resources, and decrease as the WM load increases. The amplitudes increase when cognitive resources are exclusively focused on analyzing the eliciting stimulus, and decrease when cognitive resources are consumed by other mental activity [33]. As aversive information has the ability to draw attention automatically [34,35], and the attentional resources taken up by negative emotion may no longer be available for other cognitive processes [36], it is obvious from the current results that aversive pictures as exogenous distracters take up more processing resources than neutral pictures. Hence, it is not surprising that the P3b amplitudes for both types of WM task were significantly attenuated under the negative emotion condition.

Second, the most interesting result in this study was the late sustained NSW, which also confirms our hypothesis. The retention-related NSW for the spatial tasks showed a significant negative deflection under the negative emotion condition, but that for the verbal tasks did not, indicating that the induced negative emotion selectively affected the retention function of spatial WM. This may be due to differences in the underlying mechanisms of the maintenance processes of spatial and verbal information in WM. In this experiment, the task required participants to keep the items in mind for a long period. For the spatial tasks, the three locations were probably rehearsed by shifting visual attention from location to location, whereas for the verbal tasks, the relevant information was maintained simply by phonological rehearsal. ERP studies indicate that selective attention is utilized throughout the entire period of active maintenance in spatial WM to keep relevant spatial information in mind [13,14]. In addition, negative stimuli are more capable of capturing attention than neutral stimuli [35], and the attention taken up by aversive information is known to act as a powerful exogenous cue that can result in the transient involuntary capture of spatial attention and trigger reflexive shifts in spatial attention toward its location [37,38]. Thus, visuospatial attention may overlap negative emotion and spatial WM, which can lead to affect-induced selective impairment in the retention of spatial information in WM.

Finally, the probe stimuli-locked P3b potentials revealed significant attenuated activity at the posterior poles in the negative emotion condition compared with the neutral emotion condition. In a recent study, Morgan et al. [26] employed the DMST with faces, and analyzed the ERPs in both the encoding and retrieval phases. In their study, the P3b amplitudes decreased as the WM load increased. It is thought that the P3b amplitude may be suppressed at higher WM loads because the increased cognitive demands of the task leave fewer resources available for stimulus evaluation [39]. In this study, the enhanced cognitive demands of the WM tasks in the negative emotion condition left fewer resources for the evaluation of the probe stimulus, and thus the early P3b and late P3b elicited by the probe stimulus were reduced. Both spatial and verbal WM retrieval processes were affected by negative emotion, which is thought to be the consequence of poor encoding processes of both types of WM in the target phase in the negative emotion condition.

To enable cross-reference with mental disorders, this study only included negative emotion, which leaves some questions open. Using two emotional states (negative and positive) with two types of WM (spatial and verbal) in a double dissociation design would clarify the relationship between emotion and cognition much better than the single dissociation design. It is possible that verbal WM is affected by positive emotion, but it is not possible to determine this from this study. Another issue is that the negative and neutral IAPS pictures adopted to induce emotion may have had different visual complexity and interpretability, though careful attention was paid in picking the pictures. It may still be the case that the negative pictures were more complex than the neutral pictures, thereby driving more complex visual attention processes and causing the interpretation process to be prolonged into the encoding phase of the study (and perhaps into the retention interval). If positive pictures had been included, then the effects of differences in the pictures would have been easily dissociated from the effects of emotion, as positive pictures also tend to be of high visual complexity. This is the chief limitation of this study, and has some implications for future work.

## Conclusion

In summary, this study combined a time-locked paradigm and a high-time resolution ERP technique to yield further evidence of the different underlying mechanisms of the retention processes of spatial and verbal WM. The sustained negative slow wave (NSW) revealed a significant interaction between emotional state and task type, which suggests that the negative emotion influenced the retention of spatial information because of the reliance of picture-evoked negative emotion and spatial WM on a common visuospatial attention mechanism.

## Methods

### Participants

Fifteen students (8 M 7 F, mean age = 25 ± 3.8 years) at the China Agricultural University voluntarily participated in the study. All were right-handed with normal or corrected-to-normal vision and had no previous neurological/psychiatric history. The participants received monetary compensation for their time and gave their informed consent in accordance with the IRB at the Institute of Psychology at the Chinese Academy of Sciences.

### Stimuli and Procedure

Eighty-four digitized color pictures were selected from IAPS [40]: 42 were classified as aversive pictures (e.g., mutilations, pointing guns) and 42 were classified as neutral pictures (e.g., landscapes, household appliances) (see the Additional description). In accordance with the IAPS scoring, the aversive pictures were more negative in valence than the neutral pictures (2.15 ± 0.43 vs. 5.00 ± 0.35; t (41) = -36.534, p < 0.001), and also more exciting in arousal (6.34 ± 0.62 vs. 3.24 ± 0.59; t (41) = 23.324, p < 0.001).

When presented, all of the pictures occupied between 3 and 4.5° of the angle of vision on either side of the visual midline. Each target stimulus was drawn from a set of 12 capital letters from the Latin alphabet. During the presentation, the letters on a given trial would appear at 1 of 12 positions, each of which was on 1 of 6 equidistant radii of an imaginary circular array 2 or 6 cm from the screen's center.

The participants were seated in an electrically isolated, sound- and light-attenuated room and viewed a computer monitor from a distance of 75 cm.

**Figure 5:**
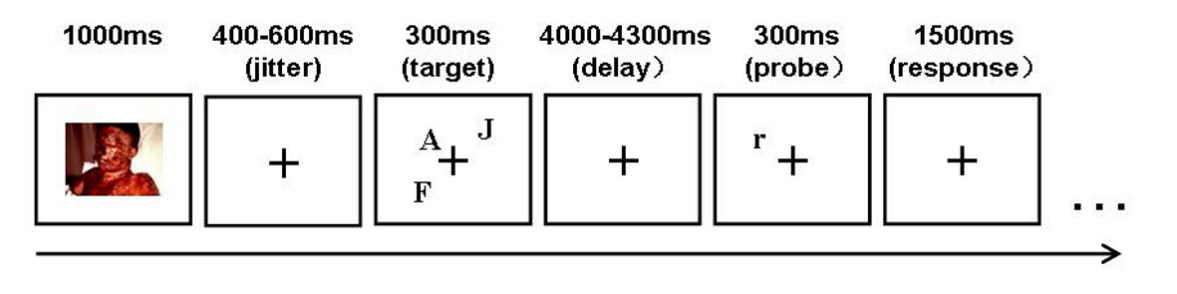
Schematic diagram of the task trails.

All of the trials began with a picture, which remained on the screen for 1000 ms, followed by a short interval (which varied randomly from 400 to 600 ms). Three capital letters at random positions around the cross hairs were then presented for 300 ms. After a short delay (which varied randomly from 4000 to 4300 ms), a lowercase letter was presented as a probe stimulus for 300 ms, and the position of this lowercase letter was also picked from the possible 12 positions (see Figure 5). The participants were asked to decide whether or not the lowercase letter was congruent with one of the preceding capital letters, and were instructed to try to respond correctly. The stimulus sets of verbal and spatial WM tasks were equivalent, but different instructions were given to the participants before the beginning of each task. In the verbal task, they were instructed to judge whether the name of the letter in the probe phase was the same as or different from the letter in the target phase, and to ignore the letter's location. However, in the spatial task, only the location of the letter, not its name, was to be remembered and judged. Half of the participants were told to press the "V" key with their left index finger for similar stimuli and the "M" key with their right index finger for different stimuli. Similar and different stimuli were presented in equal proportions. For the other half of participants, the assignment of the response hand was reversed. If no response was given, then the next trial began after 1500 ms. The participants were informed that the pictures had nothing to do with their tasks, but were asked to look at the pictures when they were presented.

The experiment was divided into two sessions that took place on the same day. At one session, only aversive pictures were presented, and at the other, only neutral pictures were presented. The sequence of aversive and neutral sessions was balanced between the subjects. This approach was used because stable emotion and WM interaction require a relatively constant emotional mood. There was a twenty minutes interval between the two sessions to avoid emotional interference. Each session included three verbal task blocks and three spatial task blocks that were pseudorandom in each session. Each block involved 24 trials, which resulted in 72 trials in each experimental condition and meant that each picture was presented at least three times (negative-verbal WM, negative-spatial WM, neutral-verbal WM, neutral-spatial WM). All of the trials in each block were automatically generated and fully randomized by the E-prime program.

Before the beginning of the experiment, the participants were given a positive and negative affect schedule (PANAS) consisting of 10 examples of positive affect (interested, excited, strong, enthusiastic, proud, alert, inspired, determined, attentive, and active) and 10 examples of negative affect (distressed, upset, guilty, scared, hostile, irritable, ashamed, nervous, jittery, and afraid) [41]. They were asked to rate how accurately each of the terms on the scale described the way that they were feeling at that moment (1 = not at all, 5 = very much). After each session of the experiment, the participants filled out another PANAS scale.

### ERP Recordings and Analysis

The electroencephalogram (EEG) was recorded from 64 scalp sites using Ag/AgCl electrodes mounted in an elastic cap (NeuroScan Inc.), with the reference on the left mastoid. Vertical electrooculogram (EOG) recording electrodes were positioned above and below the left eye, and horizontal EOG recording electrodes were positioned at the outer canthi of both eyes. All electrode impedances were kept below 5 kΩ. The EEG and EOG were bandpass filtered between DC and 100 Hz and were sampled at a digitization rate of 500 Hz. To allow off-line analysis, they were digitally filtered with a 16 Hz lowpass filter. Trials with various artifacts were rejected with a criterion of ± 100 μV. The ERPs were averaged for trials with correct responses. At least 55 trials were conducted per average per condition (61 trials for negative-verbal WM, 58 trials for negative-spatial WM, 55 trials for neutral-verbal WM, and 56 trials for neutral-spatial WM).

The ERP waveforms were time locked to the onset of the target and probe stimuli, respectively. The averaged epoch for the target stimulus-locked ERP was 4400 ms, ranging from 400 ms before the onset of the target stimuli to 4000 ms after the target stimuli. The averaged epoch for the probe stimulus-locked ERP was 1200 ms, which included a 200 ms pre-probe baseline.

**Figure 6:**
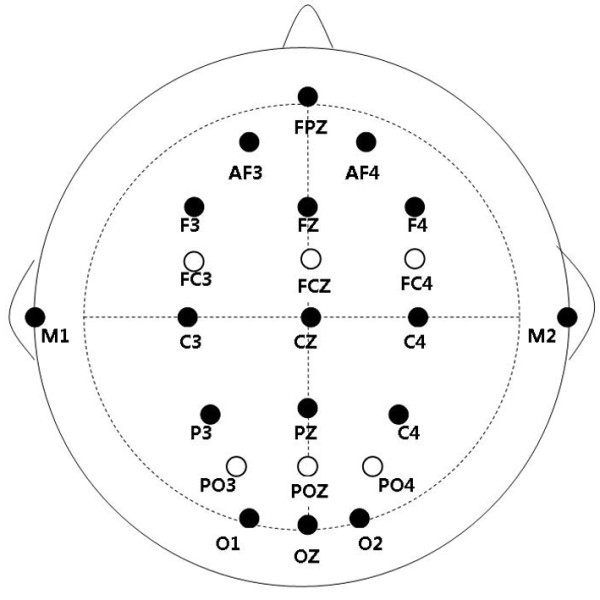
Approximate position of the 21 channels of interest selected for analysis.

The following 21 sites were chosen for statistical analysis for both the target stimulus-locked ERP and probe stimulus-locked ERP components: FPz, Fz, FCz, Cz, AF3, AF4, F3, F4, FC3, FC4, C3, and C4 (12 anterior sites); Pz, POz, Oz, P3, P4, PO3, PO4, O1, and O2 (9 posterior sites) (see Figure 6). For the target stimulus-locked ERP, the early and lately P3b potentials were measured in time windows of 280-450 ms and 330-770 ms separately over the 9 posterior and 12 anterior sites, as they had different scalp distributions. The negative slow wave (NSW) was broadly distributed over the whole scalp, and was measured by all 21 electrodes in a time window of 1000-4000 ms.

For the probe stimulus-locked ERP, the early P3b and late P3b components were mainly distributed over the nine posterior electrodes and measured in 230-460 ms and 460-800 ms time windows, respectively. To demonstrate the scalp distribution of the emotional state effects, topographic voltage maps of the difference waves (obtained by subtracting the averaged ERPs of the neutral trials from those of the negative trials) were generated at intervals of 300-600 ms.

An ANOVA of each ERP component was conducted with four factors: emotional state (negative/neutral), task type (spatial/verbal), laterality (left/midline/right), and anterior-posterior scalp location (FP/F/FC/C/P/PO/O). The Greenhouse-Geisser correction was used to compensate for sphericity violations.

## Competing interests

The authors declare that they have no competing interests.

## Authors' contributions

All of the authors read and approved the final manuscript. XL designed the study, performed the experimental work, and conceived, drafted, and edited the manuscript; RCKC commented critically on the manuscript and helped coordinate the study; YJL supervised the study design and coordination.

## Additional description

**Negative pictures were IAPS slides **2141, 2710, 2730, 2800, 2900, 3000, 3015, 3030, 3051, 3100, 3102, 3110, 3120, 3130, 3140, 3150, 3160, 3170, 3180, 3230, 3261, 3350, 6200, 6230, 6250, 6312, 6313, 6350, 6360, 6370, 6510, 6530, 6540, 6550, 6560, 6570, 6821, 6834, 6260, 6300, 9570, and 9571. **Neutral pictures were slides **1112, 1230, 1616, 2190, 2200, 2206, 2210, 2214, 2215, 2220, 2221, 2372, 2514, 2749, 2870, 2880, 2890, 5130, 5390, 5395, 5500, 5520, 5530, 5531, 5532, 5534, 5740, 7002, 7030, 7035, 7050, 7060, 7130, 7140, 7150, 7160, 7170, 7185, 7491, 7205, 7500, and 7550.
